# Genome comparison provides molecular insights into the phylogeny of the reassigned new genus *Lysinibacillus*

**DOI:** 10.1186/s12864-015-1359-x

**Published:** 2015-02-27

**Authors:** Kai Xu, Zhiming Yuan, Simon Rayner, Xiaomin Hu

**Affiliations:** Key Laboratory of Agricultural and Environmental Microbiology, Wuhan Institute of Virology, Chinese Academy of Sciences, Wuhan, 430071 China; University of the Chinese Academy of Sciences, Beijing, 100039 China; State Key Laboratory of Virology, Wuhan Institute of Virology, Chinese Academy of Sciences, Wuhan, 430071 China

**Keywords:** *Lysinibacillus*, *Bacillus*, *Lysinibacillus sphaericus*, Genome, Phylogeny

## Abstract

**Background:**

*Lysinibacillus sphaericus* (formerly named *Bacillus sphaericus*) is incapable of polysaccharide utilization and some isolates produce active insecticidal proteins against mosquito larvae. Its taxonomic status was changed to the genus *Lysinibacillus* in 2007 with some other organisms previously regarded as members of *Bacillus*. However, this classification is mainly based on physiology and phenotype and there is limited genomic information to support it.

**Results:**

In this study, four genomes of *L. sphaericus* were sequenced and compared with those of 24 representative strains belonging to *Lysinibacillus* and *Bacillus*. The results show that *Lysinibacillus* strains are phylogenetically related based on the genome sequences and composition of core genes. Comparison of gene function indicates the major difference between *Lysinibacillus* and the two *Bacillus* species is related to metabolism and cell wall/membrane biogenesis. Although *L. sphaericus* mosquitocidal isolates are highly conserved, other *Lysinibacillus* strains display a large heterogeneity. It was observed that mosquitocidal toxin genes in *L. sphaericus* were in close proximity to genome islands (GIs) and mobile genetic elements (MGEs). Furthermore, different copies and varying genomic location of the GIs containing *binA/binB* was observed amongst the different isolates. In addition, a plasmid highly similar to pBsph, but lacking the GI containing *binA/binB*, was found in *L. sphaericus* SSII-1.

**Conclusions:**

Our results confirm the taxonomy of the new genus *Lysinibacillus* at the genome level and suggest a new species for mosquito-toxic *L. sphaericus*. Based on our findings, we hypothesize that (1) *Lysinibacillus* strains evolved from a common ancestor and the mosquitocidal *L. sphaericus* toxin genes were acquired by horizontal gene transfer (HGT), and (2) capture and loss of plasmids occurs in the population, which plays an important role in the transmission of *binA/binB*.

**Electronic supplementary material:**

The online version of this article (doi:10.1186/s12864-015-1359-x) contains supplementary material, which is available to authorized users.

## Background

*Lysinibacillus sphaericus* (formerly named *Bacillus sphaericus*) is a Gram-positive, aerobic, mesophilic, and spore-forming bacterium that is commonly isolated from soil. It is also an archaic organism whose spores have even been found in 25–40-million-year-old amber [1]. *L. sphaericus* has very distinctive phenotypic properties, including an inability to utilize polysaccharide pathways and employment of exclusive metabolic pathways for synthesis of a wide variety of organic compounds and amino acids [2]. Some strains produce active insecticidal proteins against mosquito larvae, and thus have been widely used as biocontrol agents for disease-transmitting mosquitoes [3]. The mosquitocidal properties are associated with the sporulation-specific binary toxin (Bin proteins) and vegetative-specific Mtx toxins [4], as well as a novel two-component toxin (Cry48 and Cry49 proteins) produced during sporulation [5]. Compared with another mosquito pathogen, *Bacillus thuringiensis* subsp. *israelensis*, *L. sphaericus* demonstrates a higher efficiency for killing mosquito larvae and a better persistence in the field [6].

The evolutionary model and systematic classification of *L. sphaericus* continues to be debated. On the basis of flagellar agglutination, *L. sphaericus* isolates can be grouped into 49 serotypes [7]. According to DNA homology between strains, five major groups (I to V) are indicated, each probably corresponding to a separate species because of the relatively low level of homology between groups [8]. However, relatively few biochemical and morphological tests are available to distinguish *L. sphaericus* as a different species. Recently, a multi-locus sequence typing (MLST) study has indicated that the mosquitocidal strains are highly conserved and appear near-clonal [9]. This is consistent with a previous report which observed that toxic *L. sphaericus* strains are all found within DNA subgroup IIA, although in association with nine serotypes (H1, H2, H3, H5, H6, H9, H25, H26, and H48).

In 2007, *Bacillus sphaericus* was formally renamed *L. sphaericus* and, together with *Lysinibacillus boronitolerans* and *Lysinibacillus fusiformis* (formerly named *Bacillus fusiformis*), was proposed to belong to a novel genus named *Lysinibacillus* gen. nov. Since then, more and more novel isolates have been assigned to *Lysinibacillus.* The species classification was mainly based on common features in physiology and phenotype, e.g. Gram-positive, spore-forming, rod-shaped, motile, presence of the Lys–Asp type of peptidoglycan in the cell wall, the main fatty acids as iso-C_15: 0_, and the predominant menaquinones as MK-7 [10], but there is little evidence to support this classification on a genomic basis. Thus, there is a need to analyze the relationship between *Lysinibacillus* and *Bacillus* on the genomic level, and to understand the evolution of mosquitocidal *L. sphaericus*.

Although a broad spectrum of data has been collected for *L. sphaericus*, there is limited genome sequence available. One complete genome sequence is available for mosquitocidal strain C3-41 (accession numbers CP000817 and CP000818) [11], and two gapped genome sequences from reference strains KCTC 3346 (or ATCC14577) and OT4b.31(both non-toxic) have also been published [12,13]. In this study we report genome sequences of four *L. sphaericus* strains, comprising three toxic strains (2297, LP1-G, SSII-1) and one non-toxic strain (NRS1693). We also investigate their phylogenetic relationship with genome sequences for *Lysinibacillus* and *Bacillus* strains. Our results provide the first support for the taxonomy of the reassigned new genus *Lysinibacillus* at the genome level and suggest a new species for mosquitocidal *L. sphaericus*, providing new insight into the evolution of *Lysinibacillus.*

## Results

### General features

The whole genomes of *L. sphaericus* 2297, LP1-G, SSII-1 and NRS1693 were sequenced and assembled into 278, 143, 138 and 546 contigs, respectively. An additional 24 genome sequences were selected for comparison to create a final dataset of 28 genomes; 10 came from *Lysinibacillus* (seven *L. sphaericus*, two *L. fusiformis* and one *L. boronitolerans*), one from *Lysinibacillus-*related strain *Bacillus* sp. NRRL B-14905 [11]*,* and 17 from the *B. cereus* group and *B. subtilis*. The characteristics of all these genomes are summarized in Table 1.

**Table 1:** **Strains and genome information used in this study**

**Strain**	**Status**	**Genome size (bp)**	**GC content (%)**	**No. of contigs**	**No. of proteins**	**Genbank accession No.**
*B. subtilis*						
QB928	complete	4,146,839	43.60	-	4,031	NC_018520
BAB-1	complete	4,021,944	43.89	-	4,003	NC_020832
BSn5	complete	4,093,599	43.84	-	4,145	NC_014976
168	complete	4,215,606	43.91	-	4,003	NC_000964
6051-HGW	complete	4,215,610	43.51	-	4,187	NC_020507
*B. thuringiensis*						
BMB171	complete	5,330,088	35.17	-	5,352	NC_014171
Al Hakam	complete	5,257,091	35.43	-	4,798	NC_008600
IBL 200	draft	6,731,790	34.53	2	6,693	NZ_CM000758
HD-789	complete	5,495,278	35.17	-	6,462	NC_018508
Bt407	complete	5,500,501	35.02	-	6,402	NC_018877
*B. anthracis*						
Ames	complete	5,227,293	35.38	-	5,039	NC_003997
*B. cereus*						
ATCC 14579	complete	5,411,809	35.29	-	5,231	NC_004722
AH187	complete	5,269,030	35.51	-	5,783	NC_011658
E33L	complete	5,300,915	35.13	-	5,641	NC_006274
03BB102	complete	5,269,628	35.33	-	5,606	NC_012472
AH820	complete	5,302,683	35.31	-	5,810	NC_011773
biovar anthracis str. CI	complete	5,196,054	35.25	-	5,558	NC_014335
*L. sphaericus*						
C3-41	complete	4,639,821	37.13	-	4,584 (4,584)*	NC_010382
2297	draft	4,525,834	37.12	278	4,539 (4,102)*	JPDJ00000000
LP1-G	draft	4,542,839	37.20	143	4,630 (4,086)*	JPDL00000000
SSII-1	draft	4,651,985	37.01	138	4,701 (4,202)*	JPDK00000000
NRS1693	draft	4,640,690	37.55	546	4,645 (3,817)*	JPDM00000000
KCTC 3346	draft	4,560,870	37.10	83	4,443 (2,791)*	AUOZ00000000
OT4b.31	draft	4,856,302	37.51	94	4,575 (3,074)*	AQPX00000000
*L. fusiformis*						
ZB2	draft	4,550,616	37.31	59	4,494	AMQZ00000000
ZC1	draft	4,649,417	37.30	113	4,729	ADJR00000000
*L. boronitolerans*F1182	draft	4,461,358	37.49	309	5,270	AJXM00000000
*Bacillus* sp. NRRL B-14905	draft	4,497,271	37.56	99	4,470	NZ_AAXV00000000

*Number of predicted genes matched with those of *L. sphaericus* C3-41 genome.

The total genome sizes vary from 4.0 to 6.7 Mb across species and strains. All *Lysinibacillus* strains have larger chromosome sizes (4.5 ~ 4.8 M) compared to *B. subtilis* (4.0 ~ 4.2 M) but smaller sizes compared to *B. cereus* group strains (5.2 ~ 6.7 M). Conversely, their G + C content (~37%) is higher than that of *B. cereus* group strains (~35%) but lower than that of *B. subtilis* (~43%).

The numbers of predicted genes in *L. sphaericus* genomes varied from 4,470 to 4,701, but is likely a factor of incomplete genome assemblies as well as individual strain differences. With C3-41 as a reference, the predicted gene numbers of other *L. sphaericus* strains varied from 2,791 to 4,202, corresponding to 62.8 to 90.4% of the total gene numbers of the individual genome. The novel strains presented in this study (2297, LP1-G, SSII-1 and NRS1693) harbor over 80% genes predicted to be homologous to genes in C3-41, whereas the corresponding numbers in the two *L. sphaericus* reference strains KCTC3346 and OT4b.31 were only 62.8% and 65.4% respectively.

### Phylogenetic relationship

**Figure 1:**
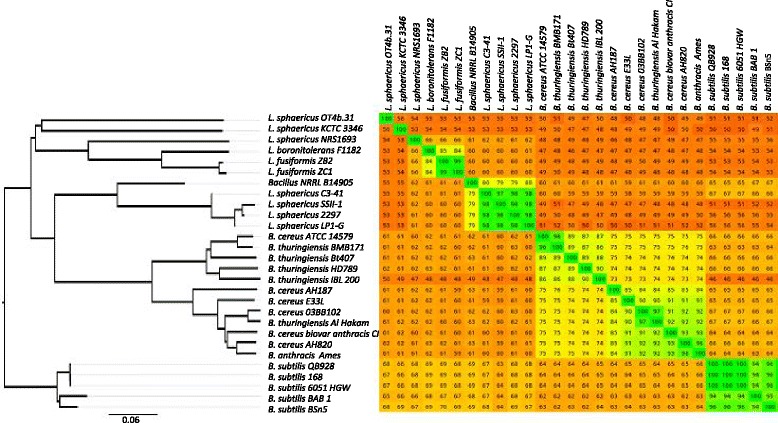
**Gegenees analysis of genome composition of 28 genome sequences (10** ***Lysinibacillus*** **, one from** ***Lysinibacillus*** **-related strain Bacillus sp.** NRRL B-14905, and 17 from *B. cereus* group and *B. subtilis*, See Table 1 for full details). Right: heat map showing pairwise comparison of each genome pair based on similarity of fragments generated by sliding window. Plot colors reflect the similarity, ranging from low (red) to high (green). The heatmap is asymmetric because the contents of genomes differ in sizes and a similarity is calculated as a fraction of similar sequences in each genome. Left: SplitsTree dendrogram using the Nexus file exported from Gegenees. The toxic *L. sphaericus* strains form a single well defined tight cluster in both the heatmap and the dendogram (green square towards the top left of heatmap), and are distinct from other strains. The scale bar represents a 6% difference in average BLASTN score similarity.

The Gegenees software package [14] was used for the comparative analysis of the gene content of the 28 genomes. The software resolves each genome into a series of overlapping fragments and then performs pairwise comparison of each fragmented genome. In this way, a distance matrix based on shared fragments is created. A heatmap of the calculated similarity matrix is shown in Figure 1. A number of genomes are well clustered, in particular the toxic isolates of *L. sphaericus* are highly conserved with >97% conservation between 2297, LP1-G, SSII-1 and C3-41 (green square towards the top left of the heat map in Figure 1), and clearly distinct from the non-toxic *L. sphaericus* isolates NRS1693, KCTC_3346 and OT4b.31 (extreme top right in the heatmap). The marine Bacillus spp. NRRL B-14905 isolate showed 79.5% similarity with the toxic isolates and 55-62% similarity with the non-toxic strains. This suggests that this marine strain has a taxonomic status that is somewhere between the toxic and non-toxic strains, but closer to the former. In addition, *L. fusiformis* and *L. boronitolerans* are related with a similarity of 84%.

Based on the distance matrix Nexus file exported from Gegenees, a dendrogram was produced using SplitsTree 4 (using the neighbor joining method) (Figure 1 left). The tree classifies all *Lysinibacillus* genomes into two main clusters. The *L. sphaericus* toxic isolates and the marine Bacillus spp. NRRL B-14905 are clustered and closer to *B. cereus* group strains, whereas the three non-toxic *L. sphaericus* strains are clustered with *L. fusiformis* and *L. boronitolerans*. Thus the *L. sphaericus* strains are diverse and scattered at the genomic level.

In addition, the genomes of *Solibacillus silvestris* [GenBank: NC_018065]*, Sporosarcina pasteurii* [GenBank: AYOX00000000]*,* and *Ureibacillus thermosphaericus* [GenBank: AJIK00000000]*,* which are thought to be sphaericus-like organisms close to *L. sphaericus* based on 16 s rDNA and phenotypic analysis [15] were investigated. The results showed that these sphaericus-like organisms were quite divergent at the genome level and there is no obvious relationship with *Lysnibacillus* and *Bacillus* (data not shown).

### Core conserved genes consensus tree

**Figure 2:**
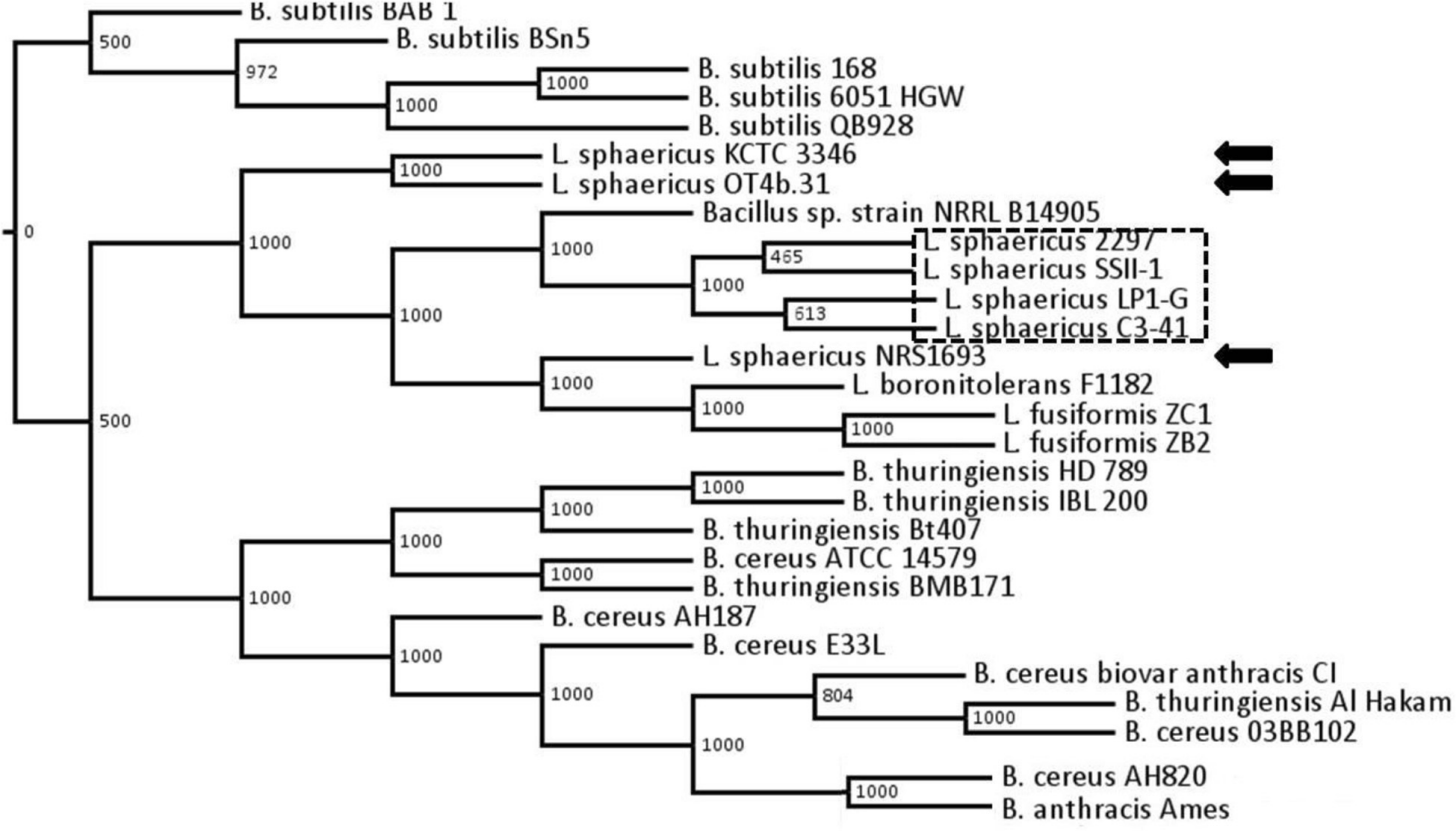
**Neighbour-joining tree showing the phylogenetic relationships among 28 strains.** NJ tree is based on 55 core genes present in all genomes. The genomes are grouped into three main clusters (1) 10 *Lysinibacillus* strains and one *Bacillus* spp, (2) *B. subtilis* and (3) *B. cereus* group. Within the *Lysinibacillus* / *Bacillus* spp cluster, the toxic *L. sphaericus* strains form a single well defined tight cluster (marked with box with dotted line) whereas the non toxic strains (marked with arrows) are less well defined. Support for clades was assessed using 1,000 bootstraps.

As a second estimate of the evolutionary relationship amongst the selected genomes, 55 core genes identified by BLAST analysis (e-value ≤ 1e-10, identity ≥ 0.75, coverage ≥ 0.75) (See Additional file 1: Table S1) were used to generate a consensus phylogenetic tree using the NJ method (Figure 2). Consistent with the previous results, all the 10 *Lysinibacillus* strains and *Bacillus* spp. NRRL B-14905 were grouped into one cluster, and the toxic *L. sphaericus* strains and *L. fusiformis* and *L. boronitolerans* each formed well supported subclusters. However, the non-toxic *L. sphaericus* strains fail to cluster and are scattered within the *Lysinibacillus* clade.

### Gene content of pan- and core genomes

**Figure 3:**
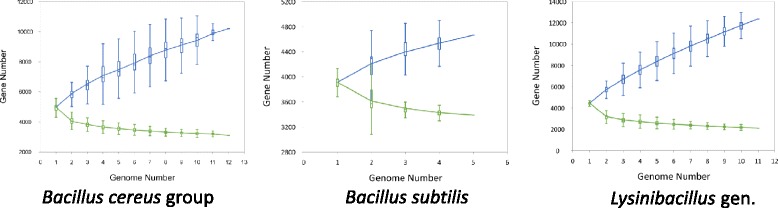
**Pan- and core genome plots. (a)** *B. cereus* group, **(b)** *B. subtilis* and **(c)** *Lysinibacillus* genomes. The blue (upper) and green (lower) curves represent pan- and core genomes respectively. Each pan- or core genome was identified using permutations of strains of each species.

To gain further insight into the relationship between the members of *Lysinibacillus* and *Bacillus*, the pan- and core genomes, which provides a measure for the intra-species variation in gene content, were each calculated using the PanGP software package [16,17]. Since the results above indicate that the *B. cereus* group and *B. subtilis* are not closely related, their pan- and core genomes were estimated individually. The resulting plots are shown in Figure 3 and highlight the differences amongst these three groups. The largest difference between the pan- and core genome is seen in *Lysinibacillus*, with the largest pan-genome (12,365) and the smallest core genome (2,113), indicating the high diversity of the genome set. The *B. cereus* group contains 12 genomes and shows the largest gene number (4,736 ~ 6,693), but possesses a smaller pan-genome (11,069) and larger core genome (3,030) compared to *Lysinibacillus*. The *B. subtilis* genomes displays the smallest difference between pan (4,666) and core genome (3,387).

**Figure 4:**
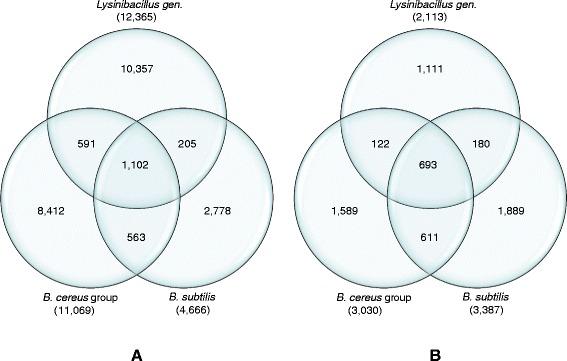
**Overlap and differences of pan and core genomes amongst the full genome set.** Venn diagrams show the overlap and difference between the **(A)** pan-genome and **(B)** core genome amongst *Lysinibacillus*, *B. cereus* group and *B. subtilis*.

In the pan-genomes, the shared genes between *Lysinibacillus* and the *B. cereus* group (1,693) is greater than the number of genes shared between *Lysnibacillus* and *B. subtilis* (1,307) or between *B. cereus* group and *B. subtilis* (1,675). For the core genome, the shared genes between *B. cereus* group and *B. subtilis* (1, 304) is much more than between *Lyninibacillus* and the other two *Bacillus* species (815 and 873, respectively) (Figure 4).

### Function features of the pan- and core genomes

**Figure 5:**
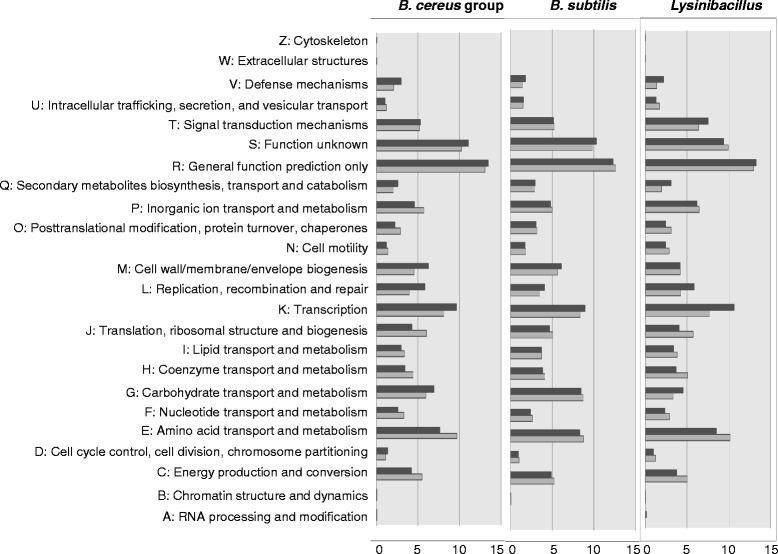
**Clusters of Orthologous Groups** (**COG) analysis of pan- and core genomes of** ***Lysinibacillus*** **,** ***B. cereus*** **group and** ***B. subtilis*** **.** COG grouping was determined according to NCBI annotation of identified proteins. Dark bars indicate the proportions of the orthologous genes assigned by COG category in the pan-genomes, and gray bars indicate corresponding proportions in the core genomes.

To investigate the functional characteristics of the pan and core genomes, the COG (Clusters of Orthologous Groups) database was used to investigate the distribution of pan and core proteins mapping to each COG category for each species group. A plot of protein proportion versus COG function by species/group is shown in Figure 5. The primary differences are observed in COG categories related to metabolism. For category G (carbohydrate transport and metabolism) the pan- and core genomes sort by protein proportion in the order *B. subtilis* > *B. cereus* group > *Lysinibacillus*. Conversely, for category E (amino acid transport and metabolism) the order is reversed, with the largest proportion observed in *Lysinibacillus*, followed by the *B. cereus* and *B. subtilis*. For the remaining classifications, the distributions of category C (Energy production and conservation), F (Nucleotide transport and metabolism), H (Coenzyme transport and metabolism), and Q (Secondary metabolites biosynthesis, transport and catabolism) both the pan- and in the core genomes of *Lysinibacillus*, were observed to be similar to those of the *B. cereus* group, but different to *B. subtilis*. A shift was observed within a genus or species for the core genome compared to the pan genome with a slight overrepresentation of COG categories related to metabolism, except G, Q (Secondary metabolites biosynthesis, transport and catabolism), and P (Inorganic ion transport and metabolism). This indicates that the gene content for metabolism of amino acids, nucleotides, coenzymes, and lipids is more conserved than for carbohydrates, secondary metabolites and inorganic ions.

Differences in the distribution of the COG categories involved in cellular process and signaling were also observed. For instance, *Lysinibacillus* displays larger proportions for category T (Signal transduction mechanisms) and N (Cell motility) both in the pan- and in the core genome compared to the *B. cereus* group and *B. subtilis*. Also, *Lysinibacillus* harbors the smallest distribution of category M (Cell wall/membrane biogenesis) features, which is almost identical in its pan- and core genome, whereas a shift was observed in *B. cereus* group and in *B. subtilis*, with a slight overrepresentation in the pan genome compared to the core genome.

A subset of COG proteins that were unique in both the pan and core genome of *Lysinibacillus* were also identified (Additional file 2: Table S2) which is probably related to species-specific characteristics. For instance, six proteins were related to ethanolamine utilization, two proteins were associated with the carbon dioxide concentrating mechanism, six were involved in cobalamin (vitamin B12) biosynthesis, one was related with the cell mobility and one with chromosome segregation.

### Characterization of gene contents of *Lysinibacillus* strains

Pairwise comparison of the genomes of all the *Lysinibacillus* strains indicate a strong syntenic relationship with *L. sphaericus* C3-41 (Additional file 3: Figure S1), indicating that *Lysinibacillus* strains may have shared a common “chromosome backbone” in a very ancient stage.

The unique genes in the 11 *Lysinibacillus* strains, varying from 34 to 711, were COG categorized (data not shown), and appear to reflect observed functional diversity for each strain. For instance, OT4b.31 displayed a large number of unique genes encoding proteins which may be related to its tolerance for heavy-metals, e.g. Co/Zn/Cd/Mg/Ni cation transporters (6 genes), metal-dependent hydrolases (3 genes), membrane proteins related to metalloendopeptidases (3 genes), Zinc metalloprotease, Mn-containing catalase, Fe-S cluster formation, and other related Oxidoreductases. KTCC 3346 contained 19 unique genes related to cell wall/membrane/envelope biogenesis, which may be associated with its ability to produce specific surface layer proteins [18-20]. It was also interesting that a gene homologous to the virion core protein of lumpy skin disease virus was identified in the marine strain *Bacillus* sp. B14905. In addition, genes encoding unique bacteriophage related proteins were identified in *L. sphaericus* 2297 (4), C3-41 (3), SSII-1 (4), OT4b.31 (11), and KCTC 3346 (7), indicating the presence of different bacteriophage(s) or prophage remnants. However, it should be noted that these data are not exact since, with the exception of C3-41, the genomes are not completely sequenced.

A previous study showed that many strains of *L. sphaericus* produce restriction endonucleases which could form a barrier to genetic manipulation [21]. The restriction enzymes and DNA methyltransferases (R-M systems) of the 11 *Lysnibacillus* strains were predicted by REBASE (http://rebase.neb.com). The result showed that the R-M systems in the *L. sphaericus* strains all belong to type II. C3-41 has the most abundant genes encoding DNA methyltransferases, with three on the chromosome and three on the plasmid pBsph, whereas 2297 and OT4b.31 only have one.

### Evolution of mosquitocidal *L. sphaericus*

**Figure 6:**
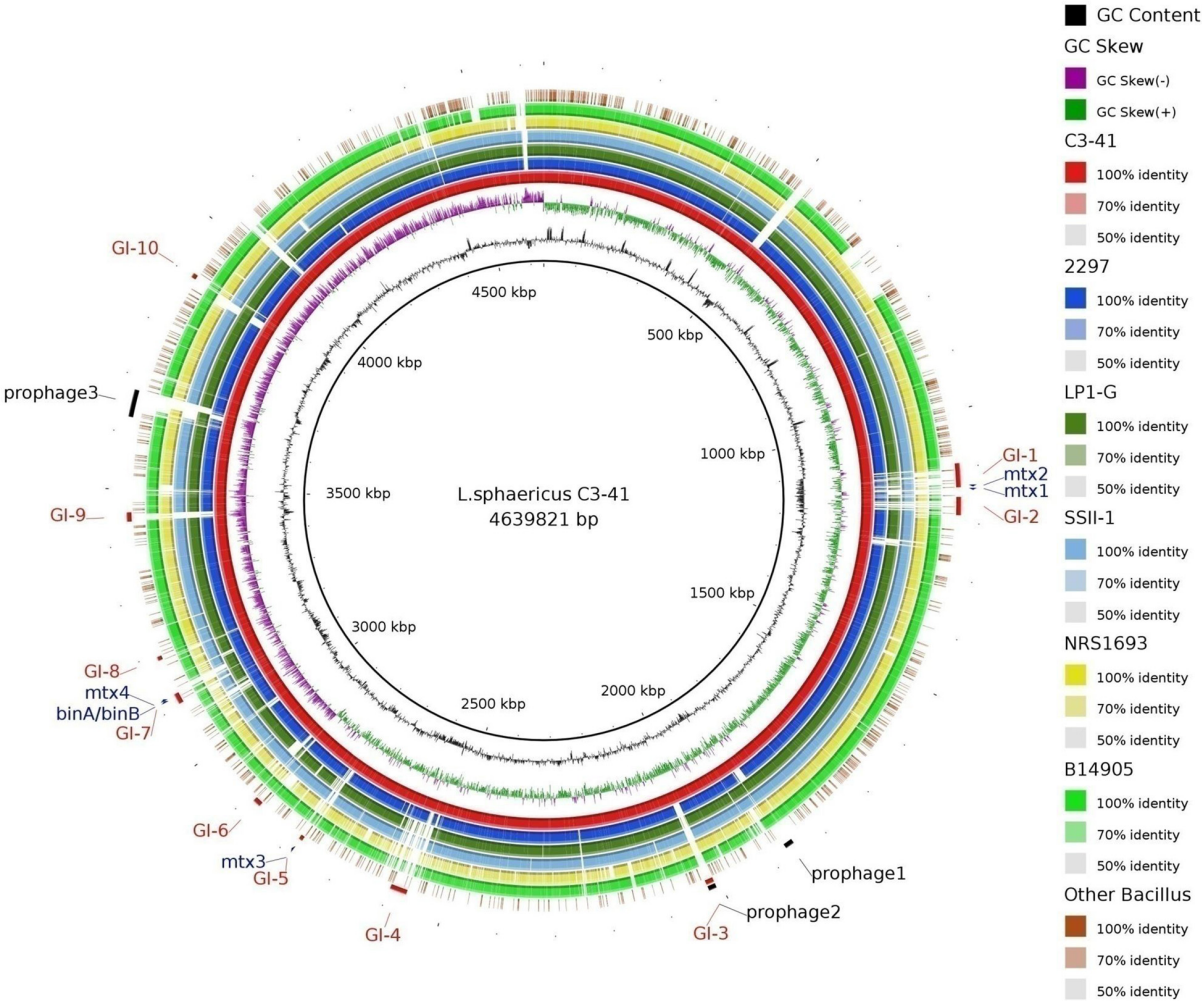
**Genome Island (GI) prediction comparative analyses of** ***L. sphaericus*** **genomes.** The C3-41 chromosome was used as reference. From the inside: circle 1, genome scale;circles 2 and 3, GC content and GC skew; circles 4–9, genome of C3-41 (red), 2297 (blue) , LP1-G (green), SSII-1 (sky blue), NRS1693 (yellow) and B14905 (emerald green), with colors from dark to light reflecting the similarity from high to low; circle 10, representative genomes for other *Bacillus* strains (i.e. *B. anthracis* strain Ames, *B. cereus* strain AH187, *B. thuringiensis* strain BMB171, and *B. subtilis* strain 168) used as outgroups and displaying similar mapping. The predicted GIs, prophages and toxic genes are marked on the outside of the circles.

10 genomic islands (GIs) were predicted in the chromosome genome of *L. sphaericus* C3-41 (Table 2, Figure 6), which are mainly located in the most hypervariable regions of the genome, and carry mobile genetic elements (MGEs), such as prophages and transposons, suggesting that these regions are associated with horizontal gene transfer (HGT). It was observed that all the mosquitocidal toxin genes are within (e.g. *mtx2/mtx3* and *binA/binB*), or close to (e.g. *mtx1*) the GIs; furthermore, these toxin genes are flanked by MGEs as previously described [11]. Thus, one possibility is that these mosquitocidal toxin genes were transferred to the common ancestor of *L. sphaericus* through HGT. GI7 (ca. 35 kb) consists of binary toxin genes *binA* and *binB*, which is the primary genetic basis of the mosquitocidal activity of *L. sphaericus*; this GI was present in C3-41, 2297 and LP1-G. A previous study showed that there are two copies of GI7 in *L. sphaericus* C3-41, present in both the chromosome and pBsph [11]. However, only one copy of GI7 was found in 2297 and LP1-G. Also, whereas C3-41 has an insert element (named ISBsph9) located downstream of *binA/binB* within GI7, a probable transposase pseudogene is presented in the equivalent region of 2297 and LP1-G.

**Table 2:** **Genome Islands (GIs) predicted in** ***L. sphaericus*** **C3-41**

**GIs**	**Containing ORFs**	**Major Function**	**Functional categories**
GI1	Bsph1038 ~ Bsph1073	Cell wall/membrane/envelope biogenesis, Mtx2	Fitness island
GI 2	Bsph1085 ~ Bsph1110	cell division or chromosome partitioning	Fitness island
GI 3	Bsph 1936 ~ Bsph 1953	Phage remnant	Symbiosis island
GI4	Bsph2575 ~ Bsph 2615	Multiple classes, major in information storage and processing	Fitness island
GI5	Bsph2815 ~ Bsph 2824	Mosquitocidal toxin	Pathogenicity island
GI6	Bsph2913 ~ Bsph 2922	Lipid transport and metabolism	Metabolic island
GI7	Bsph3179 ~ Bsph 3195	Mosquitocidal toxin	Pathogenicity island
GI8	Bsph3265 ~ Bsph 3275	Poorly characterized*	unknown
GI9	Bsph3521 ~ Bsph 3538	Replication, recombination and repair	Fitness island
GI10	Bsph4022 ~ Bsph 4035	Poorly characterized*	unknown

*Many CDSs have no matches to known function protein.

In addition, a large contig in the genome of strain SSII-1 has a high overlap (>70%) and similarity (>95%) with pBsph, indicating that SSII-1 harbors a pBsph-like plasmid (named pBsph-2). Gene function analysis revealed that this contains some genes involved in replication, recombination and repair, but no GI7 was observed in pBSph-2. It is interesting that the large plasmid pBSph and pBSph-2 contain five genes which are predicted to encode proteins homologous to the type IV secretion system (e.g. VirD4, VirB4, and VirB6) and one gene encoding pilus assembly ATPase, all which may be involved in conjugal transfer. However, the function of the pBsph-2 is still to be characterized.

## Discussion

*Lysinibacillus* belongs to the family *Bacillaceae*. Organisms in this genus were previously regarded as members of *Bacillus*, but their taxonomic status was changed to the genus *Lysinibacillus* in 2007 [10] and it remains for the classification to be confirmed on a genomic level. Moreover, as an important model bacterium for metabolism and mosquito control, the evolutionary model and systematic classification of *L. sphaericus* is a continual source of interest and debate. Therefore, exploring the phylogenetic relationship amongst members of the *L. sphaericus* genus in order to confirm the taxonomy of the reassigned new genus *Lysinibacillus* at the genomic level is of major importance. In this study, several novel genome sequences of *L. sphaericus* are reported, and their phylogenetic relationship with other genome sequences of *Lysinibacillus* and *Bacillus* strains are investigated.

The results showed that the genomes of all the studied *Lysinibacillus* strains and the marine strain *Bacillus* sp. B14905 show a high syntenic relationship with that of *L. sphaericus* C3-41, indicating these strains may have a common ancestor. Furthermore, the consensus trees based on the core genes and the genomic content indicated all the tested 10 *Lysinibacillus* organisms and B14905 are phylogenetically related and fall into a distinct and well defined cluster, confirming the taxonomy of the new *Lysinibacillus* genus. A previous study showed that one subspecies of *B. subtilis* is closely related with *L. sphaericus* based on 16 s rDNA analysis [22,23]. However, at the genome level, *Lysinibacillus* and *B. subtilis* are clustered separately. Moreover, it is interesting that despite being intergenus of *Bacillus,* the *B. cereus* group is not closely related to *B. subtilis*.

We also observed that a major difference between *L. sphaericus* and the two *Bacillus* species is the proportion of proteins encoded by the genome related to metabolism. This is in accordance with the observed species-specific metabolic characteristics; *Lysinibacillus* cannot utilize polysaccharides but alternatively metabolizes a wide variety of organic compounds and amino acids as an energy source [2]. This may explain our observation that, compared to *B. cereus* group and *B. subtilis*, *Lysinibacillus* has an abundance of genes for amino acid transport and metabolism but fewer and less variable genes related to carbohydrate transport and metabolism (probably due to functional degradation). It is interesting to note that all the *Lysinibacillus* strains have an ethanolamine utilization gene cluster. This could be a complementary pathway for an insect pathogen unable to use polysaccharide for surviving in the insect gut [24]. In addition, a difference was observed in the proportion of proteins with a COG classification of cell wall biosynthesis-related proteins, with members of the *B. cereus* group displaying a larger proportion in the pan genome than that in the core genome. This is probably because some *B. cereus* group strains, *e. g. Bacillus mycoids* and *Bacillus pseudomycoids*, have a different cell wall/membrane phenotype [25]. In contrast, the cell wall biosynthesis-related proteins in the pan-genome of *Lysinibacillus* gen. strains are almost completely complimentary to the set identified in the core genome, suggesting the strains within this genus have specific and common features in their cell wall/membrane composition [10].

Amongst *L. sphaericus*, the genomes of toxic isolates are highly conserved, whereas those of the non-toxic strains are clearly variant. This confirms a recent MLST study which indicated that there is considerably more heterogeneity amongst non-toxic strains than amongst toxic ones, with the toxic strains tested appearing near-clonal [9]. This is also consistent with a previous study which showed that recombination among *L. sphaericus* strains was relatively rare compared to the rates for most species, such as the *B. cereus* group, *Campylobacter coli*, and *Listeria monocytogenes*, and suggested that mutations were largely responsible for the generation of sequence diversity in *L. sphaericus* [9]. Due to the large heterogeneity, it is supposed that the evolutionary distance and timescale of divergence between toxic and non-toxic strains of *L. sphaericus* should be large. In contrast to the lesser variation within a single species in other *Bacillus* spp., the toxic *L. sphaericus* strains may be separated from non-toxic strains and we propose a new species should be introduced.

This raises the question of how *L. sphaericus* strains obtained mosquitocidal toxin genes and evolved into a separate population. The proximity of mosquitocidal toxin genes with the GIs and the MGEs indicates a HGT origin and the structure of GI7, a pathogenicity island containing the major mosquitocidal toxin gene *binA/binB* and MGEs, provides a possible clue. GI7 possesses multiple genomic locations across the various genomes: it is present in both the chromosome and plasmid of C3-41, but is only found in the chromosome of 2297 and LP1-G, and is absent in SSII-1; furthermore, it is present in pBSph but absent in the highly similar plasmid pBSph-2. In order to assess the basic transfer potential of pBSph and pBSph-2, homologs of the T4SS genes *virB4*, *virB6*, and *virD4* that were identified to be in the transfer region of the conjugative plasmids, e.g. the Ti-plasmid from *Agrobacterium tumefaciens,* plasmid pIP501 from *Enterococcus faecalis*, and plasmid pAW63 from *B. thuringiensis* [26,27], were investigated. The result showed that each harbor five T4SS genes displaying low levels of homology to known T4SS genes, making it doubtful that they could function as the concerted secretion machinery required for conjugation. The conjugative and transfer promoting capacities of pBsph and pBsph-2 were assessed by tri-parental matings as previously described [28]. None were indicative of self-conjugative or mobilizable activities, at least under the conditions used in the assay (detection limit of 10–7 T/R) (data not shown). One interpretation of these results is that the ancestral form of the plasmid was conjugative and genetic drifts in subsequent lineages lead to the loss of transfer capability.

A previous study surveyed the presence of toxin genes and the associated mosquitocidal activities of *L. sphaericus* isolates. It showed that non-toxic strains contain only *mtx2* or no toxin gene at all; low toxicity strains possess *mtx1*, *mtx2* and *mtx3*; and moderately or highly toxic strains contain *mtx3, binA/binB* and/or *cry48Aa/cry49Aa*, in which some isolates also contains *mtx1* and *mtx2* [9]. In addition, *mtx2* and *mtx3* are homologous and have close orthologs in *Bacillus* sp. strain NRRL B-14905 [11]. It is also interesting that Mtx2 and Mtx3 are members of *Clostridium* epsilon toxin ETX/MTX2 family (pfam 03318) of pore forming toxins defined in the NCBI Conserved Domain Database [29]. Combining the results of our analysis with these other findings, we propose the following hypothesis for the evolution of mosquitocidal *L. sphaericus*: 1) *Lysnibacillus* strains share a common ancestor; 2) A *mtx2* or *mtx3* ortholog was initially acquired by HGT; 3) The acquisition of *mtx2/mtx3* was followed by acquisition of *binA/binB*, *cry48a/cry49a* and *mtx1* also by HGT at a later time; 4) The GI containing *binA/binB* was obtained by phage integration into the chromosome and/or plasmid; 5) The ancestral form of pBsph and pBsph-2 was conjugative, whose capture and loss probably occurred in the population, probably playing an important role for the transmission of *binA/binB*. However, while the data collected to date supports this hypothesis, additional *L. sphaericus* genomes are needed together with complementary experimental and bioinformatics analysis.

## Conclusions

We present the genome sequences of four *Lysinibacillus* strains and investigate their phylogenetic relationship to other available *Lysinibacillus* strains based on analysis of genome structure and identified core genes. Our results provide the first support at the genome level for the classification of these strains into a separate genus. Our analysis also indicates that mosquitocidal *L. sphaericus* isolates appear distinct from other *Lysinibacillus* organisms at the genome level, suggesting they should be classified into a separate species. Based on our findings, we hypothesis that *Lysnibacillus* strains evolved from a common ancestor, and the mosquitocidal toxin genes were acquired by horizontal gene transfer (HGT) resulting in the evolution of the mosquitocidal *L. sphaericus*.

## Methods

### Genome sequencing

Genome sequencing of *L. sphaericus* 2297, LP1-G, SSII-1 and NRS1693 was carried out using an Illumina HiSeq 2000 system by Encode Genomics Bio-Technology Co. (Suzhou, China). Paired-end reads with average length 72 and minimum read quality of 35 were used for assembly using the Velvet-1.0.14 software package [30]. Using the genome sequence of *L. sphaericus* C3-41 [GenBank: CP000817 and CP000818] as reference, strains 2297, LP1-G and SSII-1 showed ~91% coverage, and their assembly produced 278, 143 and 138 contigs respectively. Strain NRS1693 showed ~74% coverage, and the assembly produced 546 contigs (Table 1).

### Selection of genomes used in this study

All the 10 *Lysinibacillus* genomes available at the time of analysis and one *Lysinibacillus-*related strain *Bacillus* sp. NRRL B-14905 [11] were included. The selection of 20 genomes from two representative species of *Bacillus*, *B. subtilis* and *B. cereus* group, was based on a previous study [23], which showed that *B. subtilis* is classified into two subspecies and one is closely related with *L. sphaericus*, and that *B. cereus* group is located on a clade neighboring *L. sphaericus/B. subtilis*. Thus, the five selected genomes of *B. subtilis* were well representative of the two subspecies. Since the seven members (i.e. *B. cereus*, *B. thuringiensis*, *B. anthracis*, *B. weihenstephanensis*, *B. mycoides, B. pseudomycoides* and *B. cytotoxicus*) of *B. cereus* group share close genetic and biochemical relatedness, only 15 genomes of the three major members (i.e. *B. cereus*, *B. thuringiensis*, *B. anthracis*) were selected as representative of the strains and species, other closely related or derivative strains were not included. In summary, a total of 17 complete and 11 gapped genomes from *Lysinibacillus*, *B. cereus* group, and *B. subtilis* strains were selected for analysis in this study (Table 1).

In addition, the genomes of *Solibacillus silvestris* [GenBank: NC_018065]*, Sporosarcina pasteurii* [GenBank: AYOX00000000]*,* and *Ureibacillus thermosphaericus* [GenBank: AJIK00000000]*,* which are thought to be sphaericus-like organisms close to *L. sphaericus* based on 16 s rDNA and phenotypic analysis and previously thought belong to Bacillus [15] were also selected to compare with *Lysinibacillus* strains.

### Genome annotation

Genome annotation was performed using the xBASE web service (*http://www.xbase.ac.uk/annotation/*), which comprises the following steps: (i) Glimmer is used for gene prediction; (ii) tRNA genes are predicted using tRNAScan-SE [31]; (iii) ribosomal RNA genes are searched for with RNAmmer [32]; (iv) protein BLAST is run using the translated coding sequences as a query against the reference sequence; (v) the best result for each BLAST search is imported as the gene annotation (if under the user-supplied E-value cutoff) [33,34]. Primary parameters were set as default, which sets the minimum length of a gene to be 90 bp, while the permitted maximum overlap of two genes is 50 bp, and the BLAST e-value cutoff is1e-10.

Each annotated protein was then compared to the COG database using BLASTP to identify its member functional groups.

### Fragmented alignment of multiple genomes and phylogenomic relationship

A all-against-all fragment comparison analysis was performed using Gegenees (version 1.1.5) software by fragmenting genomes and comparing all pieces with all genomes [14]. The heat-plot was based on a fragmented alignment using BLASTN with settings 500/500. The cutoff threshold for non-conserved material was 30%. A dendrogram was produced in SplitsTree version 4.12.8 (using the neighbor-joining method) made from a Nexus file exported from Gegenees [35].

Ultra-fast alignments of all *Lysinibacillus* genomes were finished by the MUMmer program (version 3.0) and the colinearity relationship of each draft genome with C3-41 was calculated [36,37].

### Pan- and core genome analysis

The respective pan- and core genomes of 12 *B. cereus* group strains, 5 *B. subtilis* strains and 11 *Lysinibacillus* strains were calculated using the PanGP software package (http://pangp.big.ac.cn) [16,38], and a BLAST Matrix was constructed using a cutoff of 1e^−10^, and 50% identity and coverage. An R-script was used to analyze the COG protein composition in the pan- and core genomes, and the results were visualized in a bar chart [39].

### Gene Islands (GIs) prediction

The GIs in the chromosome of *L. sphaericus* C3-41 were predicted using IslandViewer (*http://www.pathogenomics.sfu.ca/islandviewer/query.php*) [40]. Using the C3-41 chromosome as the reference, the draft genome sequences of 2297, LP1-G, SSII-1, NRS1693 and *Bacillus* sp. NRRL B-14905 were compared and mapped with BRIG (version 0.95) [41] and GBrowse (version 2.49) [42,43], with the complete genomes of *B. anthracis* strain Ames, *B. cereus* strain AH187, *B. thuringiensis* strain BMB171, and *B. subtilis* strain 168 as outgroups. Some distinct special sites, including the predicted GIs, prophages and the mosquitocidal toxin genes were presented graphically outside the circle map.

### Nucleotide sequence accession numbers

All four draft *L. sphaericus* genomes have been deposited at GenBank. Accession numbers are listed in Table 1.

### Availability of supporting data

The data sets supporting the results of this article are included within the article and the additional files.

## Additional files

Additional file 1: Table S1. 55 core genes conserved in *Lysinibacillus* and *Bacillus* used to construct the consensus phylogenetic tree in Figure 2. Additional file 2: Table S2. Predicted COG proteins that are both unique in the pan- and in the core genome of *Lysinibacillus*. Additional file 3: Figure S1. MUMmer analysis of the linear relationship between the genome sequence of *Lysinibacillus* strains and reference strain C3-41. The X-axis represents reference C3-41, and the Y-axis represents each strain of *Lysinibacillus*. The color bar on the right side reflects the similarity, ranging from high (red) to low (green). All contigs of draft genomes were sorted automatically with C3-41 by MUMmer.
