# Supplementary figures and images for: Genome comparison provides molecular insights into the phylogeny of the reassigned new genus Lysinibacillus
Source: BMC Genomics. 2015 Feb 27;16(1):140. doi: 10.1186/s12864-015-1359-x (PMC4363355; doi:10.1186/s12864-015-1359-x)

## Slide 1
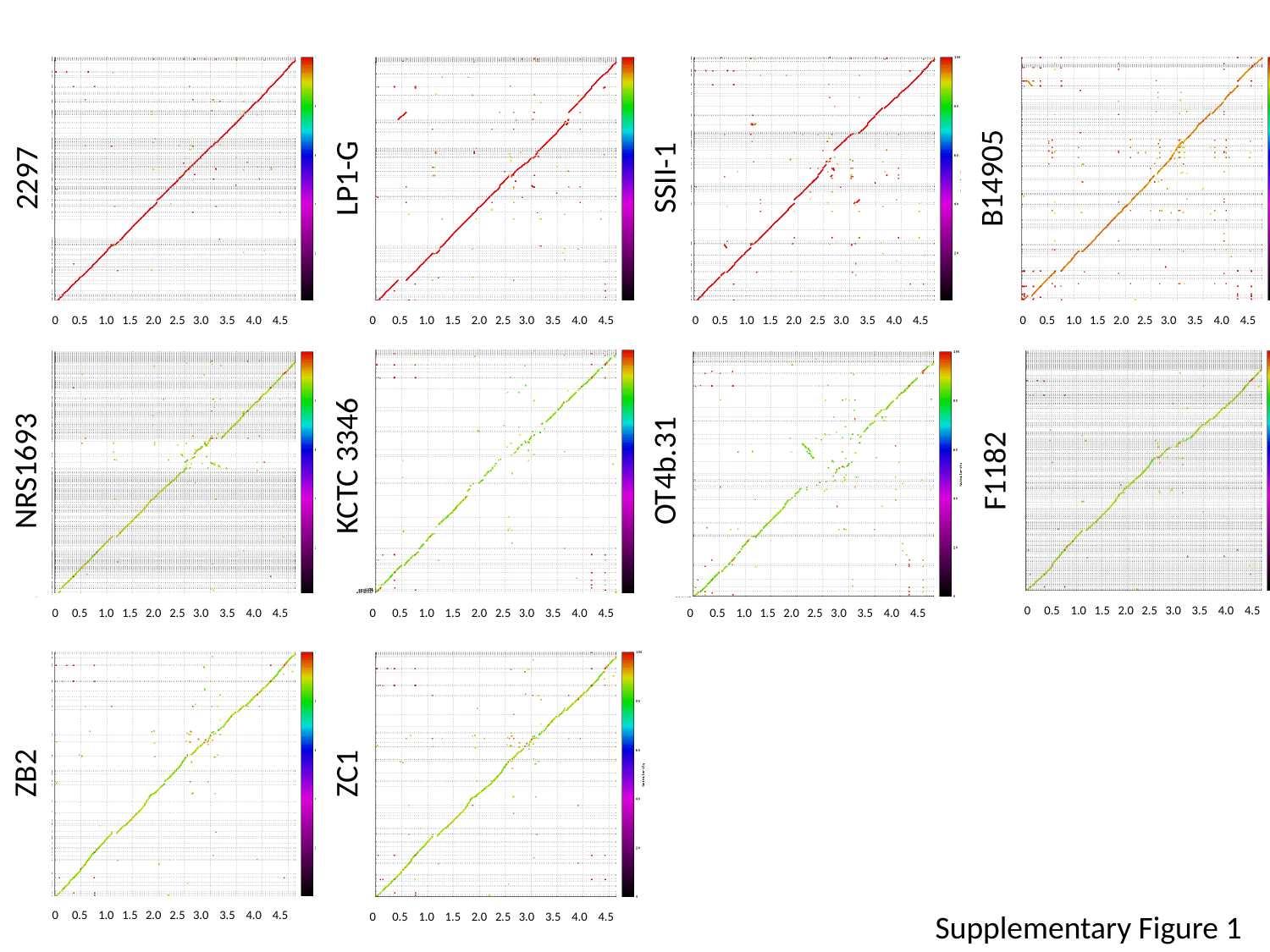

LP1-G
B14905
SSII-1
2297
0 0.5 1.0 1.5 2.0 2.5 3.0 3.5 4.0 4.5
0 0.5 1.0 1.5 2.0 2.5 3.0 3.5 4.0 4.5
0 0.5 1.0 1.5 2.0 2.5 3.0 3.5 4.0 4.5
0 0.5 1.0 1.5 2.0 2.5 3.0 3.5 4.0 4.5
KCTC 3346
F1182
OT4b.31
NRS1693
0 0.5 1.0 1.5 2.0 2.5 3.0 3.5 4.0 4.5
0 0.5 1.0 1.5 2.0 2.5 3.0 3.5 4.0 4.5
0 0.5 1.0 1.5 2.0 2.5 3.0 3.5 4.0 4.5
0 0.5 1.0 1.5 2.0 2.5 3.0 3.5 4.0 4.5
ZC1
ZB2
0 0.5 1.0 1.5 2.0 2.5 3.0 3.5 4.0 4.5
0 0.5 1.0 1.5 2.0 2.5 3.0 3.5 4.0 4.5
Supplementary Figure 1

Supplement: Additional file 3: Figure S1. — MUMmer analysis of the linear relationship between the genome sequence of Lysinibacillus strains and reference strain C3-41. The X-axis represents reference C3-41, and the Y-axis represents each strain of Lysinibacillus. The color bar on the right side reflects the similarity, ranging from high (red) to low (green). All contigs of draft genomes were sorted automatically with C3-41 by MUMmer. [file 12864_2015_1359_MOESM3_ESM.pptx]
